# Prevalence of prenatal exposure to substances of abuse: questionnaire versus biomarkers

**DOI:** 10.1186/s12978-017-0385-3

**Published:** 2017-10-25

**Authors:** Antonella Chiandetti, Gimena Hernandez, María Mercadal-Hally, Airam Alvarez, Vicente Andreu-Fernandez, Elisabet Navarro-Tapia, Adriana Bastons-Compta, Oscar Garcia-Algar

**Affiliations:** 1 Grup de Recerca Infància i Entorn (GRIE), Neonatology Unit, Hospital Clinic-Maternitat, BCNatal, C/Sabino Arana 1, 08028 Barcelona, Spain; 2 Department de Pediatria, Obstetrícia i Ginecologia i Medicina Preventiva, Universitat Autònoma de Barcelona (UAB), Barcelona, Spain; 3 Grup de Recerca en Farmacologia (GRF), Institut Hospital del Mar d’Investigacions Mèdiques (IMIM), Barcelona, Spain

**Keywords:** Prenatal exposure, Pregnancy, Questionnaire, Biomarkers, Biological matrices, Substances of abuse, Alcohol, Drugs of abuse

## Abstract

Alcohol and drugs of abuse consumption in young adults, including women of childbearing age, has experienced significant increase over the past two decades. The use of questionnaires as the only measure to investigate prenatal alcohol and drugs of abuse exposure underestimates the real prevalence of exposure and could mislead to wrong conclusions. Therefore, the aim of this article was to compare reported rates of prenatal alcohol and drugs of abuse consumption with biomarkers of exposure by a comprehensive review of the available literature. We searched MEDLINE and EMBASE databases for articles catalogued between 1992 and 2015. We identified relevant published studies that assessed the comparison between prenatal exposure to alcohol and drugs of abuse assessed by self-reported questionnaire of consumption versus biomarkers of exposure. Thirteen studies were included regarding alcohol consumption, and seven of them about drugs of abuse. Women who admitted consumption during pregnancy by questionnaire varied from 0 to 37% for alcohol, from 0 to 4.3% for cocaine, and 2.9% for tetrahydrocannabinol (THC). Positive biomarkers results ranged from 16 to 44% for alcohol, 15.4% for cocaine, and from 4 to 12.4% for THC. Biomarkers should always complement questionnaires, as it has been shown that self-report may underestimate prenatal exposure to substances of abuse.

## Resumen

El consumo de alcohol y drogas de abuso en adultos jóvenes, incluyendo mujeres en edad fértil, ha experimentado un aumento importante en las dos últimas décadas. El empleo de cuestionarios como única herramienta para detector la exposición prenatal a alcohol y drogas de abuso supone una infraestimación de la prevalencia real de la misma y podría llevar a conclusiones equivocadas. Por lo tanto, el objetivo de este estudio fue comparar las cifras declaradas de consumo prenatal de alcohol y drogas de abuso con biomarcadores de exposición mediante una revisión de la literatura. Se buscaron en MEDLINE y EMBASE artículos entre 1992 y 2015. Se identificaron los estudios publicados importantes que evaluaban la comparación entre cuestionarios de consumo autoadministrados y biomarcadores de exposición. Se incluyeron 13 estudios sobre el consumo de alcohol y 7 sobre drogas de abuso. Las mujeres que admitieron el consumo durante el embarazo por cuestionario fueron entre el 0% y el 37% para el alcohol, entre el 0% y el 4,3% para la cocaína y el 2,9% para el tetrahidrocannabinol (THC). Los valores de los biomarcadores positivos fueron entre el 16% y el 44% para el alcohol, el 15,4% para la cocaína y el 12,4% para el THC. Los biomarcadores deben acompañar siempre al cuestionario ya que se ha demostrado que la autodeclaración puede infraestimar la exposición prenatal a sustancias de abuso.

## Plain English summary

Alcohol and drugs of abuse consumption in young adults has increased over the past two decades, also in pregnant women. If we ask in a questionnaire for alcohol and drugs of abuse during pregnancy, it will be difficult to know the real consumption in most of cases. A biomarker is a substance that can be detected in the body, i.e., in blood or in hair. Therefore, the aim of this article was to compare reported consumption of prenatal alcohol and drugs of abuse consumption with biomarkers of exposure by a comprehensive review of the available literature. We searched MEDLINE and EMBASE databases for articles catalogued between 1992 and 2015. We identified relevant published studies that assessed the comparison between prenatal consumption of alcohol and drugs of abuse assessed by questionnaire or by biomarkers. Thirteen studies were included regarding alcohol consumption, and seven of them about drugs of abuse. Women who admitted consumption during pregnancy by questionnaire varied from 0 to 37% for alcohol, from 0 to 4.3% for cocaine, and 2.9% for tetrahydrocannabinol (THC). Positive biomarkers results ranged from 16 to 44% for alcohol, 15.4% for cocaine, and from 4 to 12.4% for THC. Biomarkers should always complement questionnaires, as it has been shown that self-report may underestimate prenatal consumption of substances of abuse.

## Background

Alcohol and drugs of abuse consumption in young adults and women of childbearing age has experienced an increase over the past two decades. Since nearly 50% of pregnancies are unplanned, prenatal exposure to alcohol and/or drugs of abuse in the early stages of pregnancy is relatively common. Drugs of abuse consumption rates in Spain is one of the highest in Europe, especially for cocaine and cannabis. According to the Spanish Drug Observatory latest report [1], women aged between 15 and 34 years admitted drugs of abuse consumption for the previous 12 months, 19.4, 4.3 and 1.8% for cannabis, cocaine and ecstasy, respectively.

Substances such as tobacco and alcohol show consumption patterns with a considerable high prevalence in women between 15 and 39 years old. The prevalence of alcohol consumption for the last 12 months in women from 15 to 34 years old and from 35 to 64 years old is 80.1 and 77.7%, respectively [1]. In Sweden, it has been reported that 30% of pregnant women continue the consumption of alcohol while pregnant [2]. In USA, the rate of current illicit drug use in the combined 2012–2013 data was 14.6% among pregnant women aged 15 to 17, 8.6% percent among women aged 18 to 25, and 3.2% among women aged 26 to 44. Among pregnant women aged 15 to 44 in 2012–2013, an annual average of 9.4% reported current alcohol use, 2.3% binge drinking, and 0.4% heavy drinking [3].

Prenatal exposure to these substances increases the risk of obstetric complications and has serious consequences, not only in the developing foetus, but also lifelong implications. The deleterious effects of ethanol during pregnancy are well described by the all-encompassing term ‘Foetal Alcohol Spectrum Disorder’ (FASD), which includes a wide range of physical defects, behavioural, emotional and cognitive deficits, as well as congenital anomalies [4]. Prenatal cocaine use has been associated with placental abruption and premature labour, as well as with increased rate of low birth weight, microcephaly and congenital anomalies. Gestational cannabis use is related to lack of attention, impulsivity and deficits in learning and memory. Foetal exposure to opiates has been related mainly to neonatal withdrawal syndrome and poor obstetric outcome [5].

While brain damage caused by toxics consumption cannot be repaired, we can achieve the best neurological development of these children with the early onset of follow up and, in this way, try to decrease the occurrence of secondary disabilities (poor school performance, addictions, and mental health problems) and prevent recurrence in subsequent pregnancies. Early detection of prenatal exposure to toxic substances allows these patients benefit from close monitoring of their development, treatment, early recognition of withdrawal syndrome and implement timely interventions. For this reason, identification of substance exposed infants is a key factor on preventing alcohol and drugs of abuse related birth defects. This is a major public health problem all over the world, with a severe impact on society.

There are few screening instruments to evaluate drug of abuse consumption in pregnant women and most of them have been designed to screen alcohol misuse. The most widely used measures are the following validated questionnaires: T-ACE (Tolerance, Annoyance, Cut Down, Eye Opener), TWEAK (Tolerance, Worried, Eye-openers, Amnesia, K[C] Cut Down), AUDIT and its shorter version, the C-AUDIT (Alcohol Use Disorders Identification Test), MAST and its shorter version SMAST (Michigan Alcoholism Screening Test) [6–8].

There are also lots of “local questionnaires” which include questions to screen for maternal alcohol consumption during pregnancy that are currently used like a standard tool by nursing staff [9, 10]. It is worthy of note that these questionnaires do not reflect the risk of alcohol use and show low to moderate specificity [6, 7]. Moreover, there are no specific validated questionnaires about drugs of abuse during pregnancy.

Besides the lack of specificity and sensitivity of these questionnaires, the problem of underreporting consumption by pregnant women needs to be seriously considered. Subjects may underestimate their consumption and/or are unwilling to disclose their habits during pregnancy due to fear of legal repercussions, guilt, memory biases or lack of preparation on how to perform the interview are some of the factors that can lead to minimize or deny consumption [11, 12].

For all these reasons currently, a number of biomarkers have been evaluated and are available for the purpose of detecting prenatal exposure. Maternal hair and meconium analysis are the most commonly matrices used to detect prenatal exposure, since they allow to expand the detection window of the consumption. Maternal hair provides information depending on its length (hair has a growth rate if 1 cm per month) and meconium serves as a reservoir of foetal chemical exposures during the second and third trimesters pregnancy [9, 13]. For the evaluation of alcohol intake, detection of non-oxidative direct ethanol metabolites such as fatty acid ethyl esters (FAEEs), ethyl glucuronide (EtG) and ethyl sulphate (EtS) currently appear most promising. Each of these biomarkers remain positive in maternal serum and urine for a certain amount of time after the cessation of alcohol intake (FAEEs in serum up to 24 h and EtG in urine up to 5 days) and EtG and FAEE can be detected in hair for months. Additionally, it is known that once FAEEs are formed they do not cross the human placenta; therefore if detected in meconium they represent foetal exposure to ethanol [9, 14].

In some studies, other biomarkers have been used, like carbohydrate-deficient transferring (CDT) and phosphatidylethanol (PEth) in mother blood, but they respond to regular heavy or moderate alcohol consumption in the previous 2–4 weeks [10, 15]. For the evaluation of drug consumption, the presence of cocaine (COC), benzoylecognine (BE), tetrahydrocannabinol (THC), amphetamine (AMP), metamphetamine (MDMA), opiates (OP) can be determined in maternal hair and meconium using standard chromatographic techniques. In some studies maternal urine is also used but only detects exposure for 1–4 days prior to delivery [16].

Several authors recommend universal or directed screening population of prenatal exposure to abuse substances using biomarkers. In some Mediterranean countries, although 99% of women declare absolute abstention from drinking during pregnancy, FAEEs above 2 nmol/g meconium (the cut-off internationally used to differentiate heavy maternal alcohol consumption during pregnancy from occasional or no use) ranged from 1.7% of samples in Reggio Emilia, Italy to 44.5% in Barcelona, Spain [16, 17]. In the same cohorts, meconium analysis showed that prevalence of opiates, cocaine and combined drug exposure was 8.7, 4.4 and 2.2%, whereas structured interviews only disclosed 1.3, 1.8 and 1.3% of mothers exposed to opiates, cocaine and both drugs. Clearly, in these cohorts, the usefulness of a questionnaire is absolutely futile [17].

We hypothesize that the use of questionnaires as the only measure to investigate prenatal alcohol and drugs exposure underestimate the real prevalence of exposure and could mislead to wrong conclusions. Therefore, the aim of this article was to compare reported rates of prenatal alcohol and drugs of abuse exposure with biomarkers of exposure by a comprehensive review of the available literature.

## Methods

We searched in MEDLINE and EMBASE databases for articles catalogued from 1992 to 2015 and published in English language. We identified relevant published studies that considered the comparison between prenatal exposure to alcohol and drugs of abuse assessed by validated self-reported questionnaire versus biomarkers of exposure (meconium, hair, urine, and serum). Some biological matrices including umbilical cord blood and sweat were not included since it only show very recent consumption before the collection of the sample. Metabolites of substances of abuse in it could be subrogate biomarkers of chronic consumption, but this point was not included in our analysis. We included only confirmative assays with sophisticated analytical assays, not drug screening assays, i.e. in urine. A positive assay in urine needs a confirmative assay with gas/liquid chromatography-mass spectrometry (GC-MS or LC-MS) because of the risk of false positive results. A narrative review was carried out searching combinations of key words “pregnancy” AND “substances of abuse” OR “prenatal exposure” AND “questionnaire” OR “biomarkers” OR “biological matrices” The major inclusion criteria was “diagnosis/identification/detection of prenatal exposure to drugs of abuse or alcohol”. The exclusion criteria were not to meet all inclusion criteria. Data were extracted by the authors in cooperation with bibliography managers from the university and the hospital library.

## Results

**Fig. 1:**
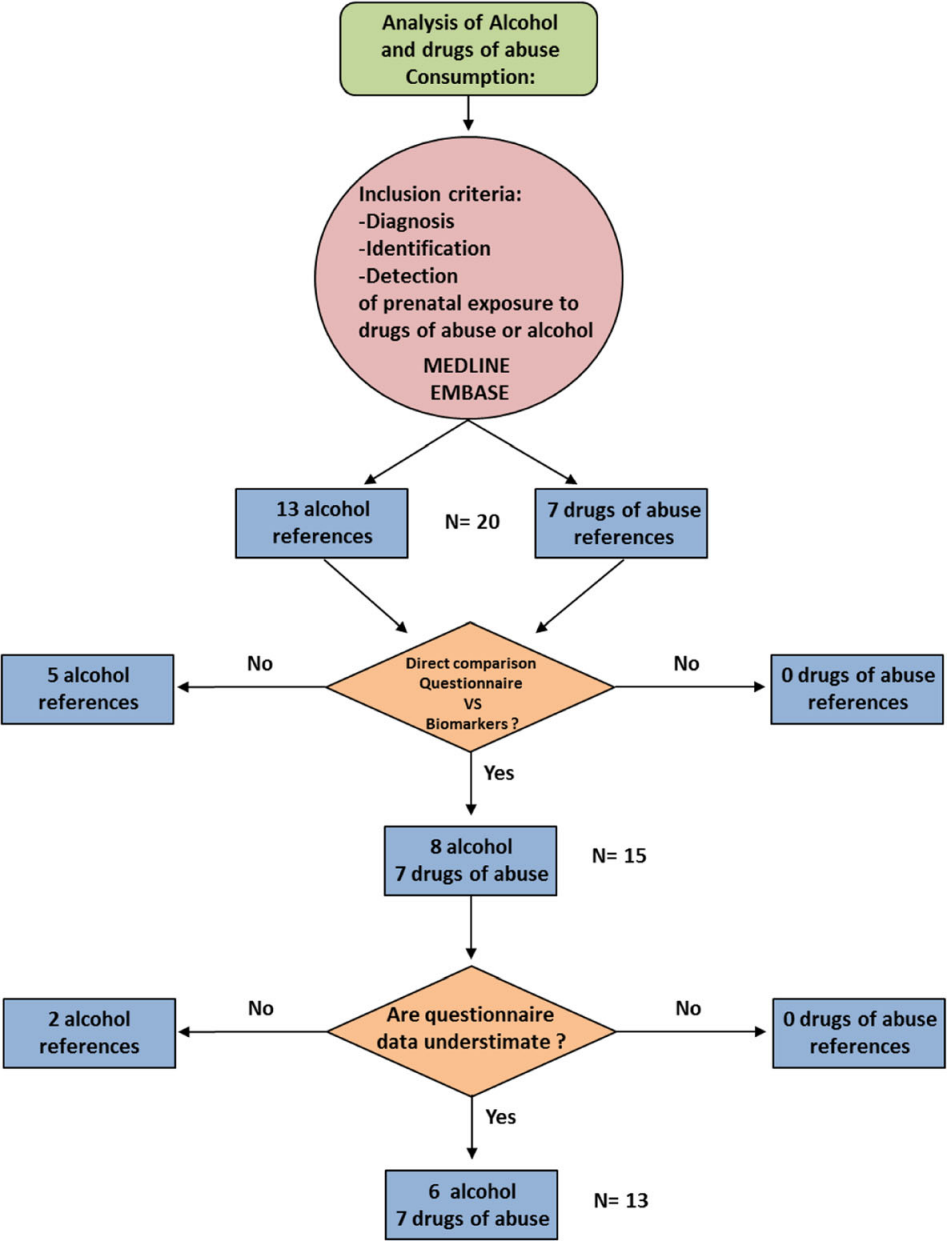
The flow diagram shows the screening process of retrieved articles, including the number and reason of exclusion

The search strategy generated 13 references regarding alcohol consumption for the final analysis (Fig. 1). The main objectives in 8 of these studies were to compare self-reported ethanol intake with the detection of biomarkers. In the other five, the objective was to estimate the prevalence of alcohol consumption using biomarkers and questionnaires although, as endpoints, they also compared both screening methods. The number of patients included varied widely between 51 and 1700 [18, 19]. The matrices used were meconium, hair [14, 18], urine [14] and serum [10, 15]. The main biomarkers used were EtG and FAEE. On the other hand, the questionnaires used varied importantly between studies (CAGE, AUDIT, CUAL, Parkyn Screening tool and PAU or prenatal alcohol use interview) (Table 1).

**Table 1:** Characteristics of included studies (alcohol)

ALCOHOL					
Author (reference, year, country)	Objective	Sample size	Questionnaire	Matrix Biomarker- Method (cut-off)	Comments
Budd et al. (10) (2000) (Ohio)	To compare the sensitivity and specificity of PAUI and ACOG antepartum record and compare their levels of CDT (recent heavy drinking)	N(BQ) = 56	1. PAUI (Prenatal Alcohol Use Interview) 2. ACOG record (contains information about past and current pregnancy and medical history)	Maternal Blood CDT - LC	
Derauf et al. (24) (2003) (Hawai)	To assess the concordance between maternal self-reported ethanol intake and detection of FAEE	N(B) = 422 N(Q) = 436	Structured questionnaire for gestational alcohol consumption	Meconium FAEE – GC-MS (50 ng/g)	
Garcia Algar et al. (31) (2008) (Spain)	To determine the prevalence of ethanol consumption	N(BQ) = 353	Self reported use	Meconium FAEE - LC-MS/MS (600 ng/g)	Self reported method not described
Gareri et al. (19) (2008) (Ontario)	To establish an objective foetal ethanol exposure prevalence using meconium FAEE and COMPAR€ with results obtained by postpartum questionnaire	N(B) = 695 N(Q) = 1019	2. Parkyn Screening Tool: standard post partum questionnaire	Meconium FAEE - GC-MS (2 nmol/g)	
Wurst et al. (14) (2008) (Sweden)	To evaluate whether biomarkers of alcohol consumption provide additional information compared with the use of a validated questionnaire	N(BQ) = 109	AUDIT (*)	Urine EtG – LC-MS EtS – LC-MS (0.05 mg/L) Hair FAEE – GC-MS (0.7 pg/mg) EtG – GC-MS (>7 pg/mg: social drinker;> 25 pg/mg excessive and repeated alcohol drinkers)	
Pichini et al. (23) (2009) (Italy, Spain))	To evaluate of two new biomarkers of exposure of alcohol (EtG and EtS) Comparison between them, with FAEEs	N(BQ) = 177 (Italy: 96; Spain: 81)	Structured questionnaire for gestational alcohol consumption	Meconium FAEE – LC-MS/MS (2 nmol/g) EtG – LC-MS/MS (5 ng/g) EtS – LC-MS/MS (1 ng/g)	Not clear how was the questionnaire
Bakdash et al. (21) (2010) (Denmark)	To compare results of FAEE and EtG on intrauterine exposure to ethanol	N(BQ) = 602	1. Structured questionnaire 2. CAGE test (*) 3. Personal interview	Meconium FAEE - GC-MS (500 ng/g) EtG - LC-MS (10 ng/g)	Sample size of questionnaire not described
Goh et al. (9) (2010) (Ontario)	To compare the prevalence of FAEE+ meconium in the general population to high risk population (high risk pregnancy)	N(BQ) = 732 (general population: 682; risk population: 50)	1. Self reported use 2. Parkyn Screening Tool: standard post partum questionnaire	Meconium FAEE – GC-MS (2 nmol/g)	
Hutson et al. (22) (2010) (Uruguay)	To determine the incidence of prenatal alcohol and drug exposure in public health care sector	N(B) = 905 N(Q) = 900	1. Structured questionnaire for gestational alcohol consumption 2. CAGE (*)	Meconium FAEE - GC-FID (2 nmol/g)	
Comasco et al. (15) (2012) (Sweden)	To evaluate methods to assess maternal drinking during pregnancy	N(BQ) = 2264	C-AUDIT (*)	Maternal Blood CDT - LC PEth - LC-MS	
Manich et al. (20) (2012) (Spain)	To compare prenatal exposure to alcohol consumption by questionnaire and biomarkers	N(BQ) = 62	Structured questionnaire for gestational alcohol consumption	Meconium FAEE - LC-MS/MS (2 nmol/g)	Questionnaire construction not clear.
Pichini et al. (25) (2012) (Italy)	To assess prenatal exposure to ethanol by measurement of EtG y FAEEs	N(BQ) = 607	AUDIT questionnaire (*)	Meconium FAEE – LC-MS/MS (2 nmol/g) EtG – LC- MS/MS (2 nmol)	
Lendoiro et al. (18) (2013) (Spain)	The aim of this work was to compare maternal interview and hair analysis to determine alcohol consumption throughout pregnancy and to study relations among maternal interview, hair results, and neonatal outcomes	N(BQ) = 51	Structured questionnaire for gestational alcohol consumption	Hair EtG – LC-MS/MS (<7 pg/mg: negative; 7–30 pg/mg: social drinker; >30: chronic drinker)	

N(B): biomarker sample size; N(Q): questionnaire sample size; N(BQ): biomarker and questionnaire sample size; (*): validated questionnaire; *GC-MS* gas chromatography-mass spectrometry, *LC-MS* liquid chromatography-mass spectrometry, *LC-MS/MS* liquid chromatography-tandem mass spectrometry, *FAEE* fatty acid ethyl esters, *EtG* ethyl glucuronide, *EtS* ethyl, *CDT* Carbohydrate-deficient transferring, *PEth* Phosphatidylethanol

The percentage of women who admitted consumption in questionnaires varied from 0% [20, 21] to 37% [22]. In the first group, positive results in biomarkers were up to 16% in both of them, whilst Hudson found up to 44% positives for FAEE. The highest positive prevalence through biomarkers was in the Spanish group led by Pichini who found up to 95% positive for EtG [23].

The main results of 11 of these studies showed how self-reported alcohol consumption is underestimated (Fig. 1). Only one of them showed how maternal interview for alcohol exposure is more sensitive than hair analysis [18]. Regarding biomarkers, Derauf et al. and Hutson et al. found no agreement between reported ethanol intake during third trimester and FAEE, with absence of FAEE in infants meconium whose mothers admitted drinking [22, 24]. Pichini et al. could not find a good correlation between FAEE, EtG and EtS [23, 25] (Table 2).

**Table 2:** Differences between questionnaire and biomarkers in included studies (alcohol)

ALCOHOL			
Author (reference, year)	Questionnaire (%positive result)	Biomarkers (%positive results)	Differences/Comments
Budd et al. (10) (2000)	PAUI (n: 56) drinkers (*n* = 26) quitters (*n* = 30) ACOG (*n* = 36) drinkers (*n* = 5) quitters (*n* = 31)	CDT n:56 High: drinkers: 32 Low: quitters: 24 N:36 High: drinkers: 21 Low: quitters: 15	- PAUI is better than ACOG record (less false negative) in order to identify drinkers
Derauf et al. (24) (2003)	Structured questionnaire (5.3%)	FAEE (17.1%)	- `No agreement between reported ethanol intake during third trimester and FAEE (absence of FAEE in the meconium of infants whose mothers admitted drinking)
Gareri et al. (19) (2008)	Parkyn questionnaire (0.5%)	FAEE (2.5%)	- Maternal screening using questionnaire would miss most of cases
Garcia Algar et al. (29) (2008)	Self-reported use (2.3%)	FAEE (45%)	- Prevalence of alcohol consumption: 45% - Underreporting of drinking
Wurst et al. (14) (2008)	AUDIT (8.7%)	EtG (0.9%) EtS (0%) (urine) EtG (15, 5%) EtS (2, 9%) (hair)	In ALL: 25.2% identified as consumers −6 only AUDIT −14 only EtG in hair - 3 only FAEE in hair - 3 both AUDIT and biomarkers The combined use identifies more subjects FAEE and EtG in hair permit to distinguish social to heavy drinkers
Pichini et al. (23) (2009)	Structured questionnaire (3.5% Italy; 4.8% Spain)	FAEE (8%); EtG (81%); EtS(46%) (Italy) FAEE(42%); EtG (95%); EtS (52%) (Spain)	- NO correlation between biomarkers and self-reporting - No direct correlation between EtG EtS and FAEE - Cut off for EtG and EtS does not exist.
Goh et al. (9) (2010)	1. Self-reported use (0%) 2. Parkyn questionnaire (1%)	FAEE (2.5%) (general population) FAEE (30%) (risk population)	- High prevalence of positive meconium among newborns in high risk obstetric unit: - Ethanol induces to perinatal risks - Chronic alcohol use in women tend to exhibit higher rate of perinatal comorbidities
Bakdash et al. (21) (2010)	1. Comprehensive questionnaire of FRAMES study (0%) 2. CAGE test (*) (1%) 3. Personal interview (0.2%)	FAEE (7.1%) EtG (16.3%)	- Optimal agreement using cut off 500 ng/g (FAEE) and 274 ng/g (EtG) - 6 mothers who answered yes in CAGE: no positive in biomarkers - The one who recognised drinking 2 glasses of wine/day: very high FAEE and EtG
Hutson et al. (22) (2010)	1. Structured questionnaire (37%) 2. CAGE (14%)	FAEE (44%)	- No correlation: the incidence would be underestimated if achieved through self-reported - Poor agreement between reported ethanol intake and FAEE (absence of FAEE in the meconium of infants whose mothers admitted drinking)
Manich et al. (20) (2012)	Structured questionnaire (0%)	FAEE (16.12%)	- Difference between self-reported and biomarkers results
Pichini et al. (25) (2012)	1. Self-reported use (56.6%)	FAEE and/or EtG (7.9%)	- No correlation between maternal self-report and results - Unspecific questionnaire, not validated
Comasco et al. (15) (2012)	C-AUDIT (12,3%)	CDT (0%) PEth (0%)	- AUDIT quick and inexpensive screening - CDT and PEth respond to regular heavy or moderate alcohol consumption in the previous 2–4 weeks
Lendoiro et al. (18) (2013)	Structured questionnaire (13.7)	EtG (3.9%)	- Hair analysis showed NOT to be more sensitive than maternal interview for alcohol exposure

Seven articles were included for the final analysis of drugs of abuse during pregnancy studies. (Figure 1) The main objective was to determine the incidence or prevalence of prenatal drug exposure in 4 of them. The other 3 studies aimed to compare biomarkers and questionnaires. The number of patients included in these studies varied from 107 to 1800 [22, 26]. The matrixes used were hair (2 studies), meconium (3 studies) or both (2 studies). These studies agreed that either meconium or hair analysis showed to be more sensitive than maternal interview for drugs of abuse. Garcia-Serra et al. found more sensitivity in hair analysis than maternal meconium to detect cannabis [16] (Table 3).

**Table 3:** Characteristics of included studies (drugs of abuse)

DRUGS OF ABUSE					
Author (reference, year, country)	Objective	Sample size	Questionnaire	Matrix Biomarker- Method (cut-off)	Comments
Garcia Algar et al. (30) (2009) (Spain)	To determine the prevalence of illegal drug use by pregnant women and subsequent foetal exposure	N(BQ) = 1209	Structured questionnaire for gestational alcohol and drugs consumption	Meconium COC - LC-MS and GC-MS (>3 ng/g) MOR- LC-MS and GC-MS (>4 ng/g) BE - LC-MS and GC-MS (>4 ng/g) THC - LC-MS and GC-MS (>20 ng/g) AMP - LC-MS and GC-MS (>5 ng/g) MDMA - LC-MS and GC-MS (>4 ng/g)	
Hutson et al. (22) (2010) (Uruguay)	To determine the incidence of prenatal alcohol and drug exposure in public health care sector	N(B) = 905 N(Q) = 900	Structured questionnaire for gestational alcohol and drugs consumption	Meconium COC - ELISA (>80 ng/g) BE- ELISA (>80 ng/g) THC - ELISA (>50 ng/g) AMP - ELISA (>100 ng/g) MDMA	
Bessa et al. (11) (2010) (Brazil)	To check the validity of the self-report of drug use by pregnant adolescents, by comparing their responses to a structured interview about their use of cocaine and marijuana during the pregnancy with an analysis of their hair	N(BQ) = 1000	Structured questionnaire for gestational alcohol and drugs consumption	Hair COC - ELISA (>0.20 ng/mg) OP - ELISA (>0.20 ng/mg) AMP - ELISA (>0.50 ng/mg) EX -ELISA (>0.50 ng/mg) all positive samples -GC-MS	
Friguls et al. (26) (2012) (Spain) García-Serra et al. (16) (2012) (Spain)	1: To estimate prevalence of drug use by pregnant women in Ibiza (Friguls) 2: To compare two biological matrices (maternal hair and meconium) to assess prenatal exposure to drugs of abuse in the third trimester of pregnancy (Garcia-Serra) 3: To evaluate the clinical applicability of these biological matrices (Garcia-Serra)	N(BQ) = 107	Structured questionnaire for gestational alcohol and drugs consumption	Hair COC - ELISA (>0.20 ng/mg) OP - ELISA (>0.20 ng/mg) AMP - ELISA (>0.50 ng/mg) EX -ELISA (>0.50 ng/mg) all positive samples -GC-MS Meconium: COC - LC-MS and GC-MS (>3 ng/g) BE - LC-MS and GC-MS (>4 ng/g) THC - LC-MS and GC-MS (20 ng/g) AMP - LC-MS and GC-MS (>5 ng/g) MDMA - LC-MS and GC-MS (>4 ng/g)	Drug exposure was defined as categorical (yes/no)
Joya et al. (32) (2012) (Spain)	To estimate prevalence of drug use by pregnant women in Tenerife Island	N(BQ) = 347	Structured questionnaire for gestational alcohol and drugs consumption	Hair COC - ELISA (>0.20 ng/mg) OP - ELISA (>0.20 ng/mg) AMP - ELISA (>0.50 ng/mg) EX -ELISA (>0.50 ng/mg) all positive samples -GC-MS	
Lendoiro et al. (18) (2013) (Spain)	The aim of this work was to compare maternal interview and hair analysis to determine drug consumption throughout pregnancy and to study relations among maternal interview, hair results, and neonatal outcomes	N(BQ) = 209	Structured questionnaire for gestational alcohol and drugs consumption	Hair COC - LC-MS and GC-MS (>500 pg/ng) MOR- LC-MS and GC-MS (>200 pg/ng) BE - LC-MS and GC-MS (>50 pg/ng) THC - LC-MS and GC-MS (>50 pg/ng) MDMA - LC-MS and GC-MS (>200 pg/ng)	

*COC* cocaine, *BE* benzoylecognine, *THC* tetrahydrocannabinol, *AMP* amphetamine, *MDMA* metamphetamine, *OP* opiates, *EX* extasis, *MOR* morphine

The percentage of women who admitted drugs of abuse consumption in questionnaires varied from 0 to 4.3% for cocaine and 2.9% for THC [11, 18]. Positive results in biomarkers were up to 4% for THC, whilst Lendorio et al. found up to 15.4% positive for cocaine and 12.4% positives for THC [18].

The collected data showed that the use of biomarkers was more sensitive than maternal interview to detect drugs of abuse consumption in pregnant women as reported with alcohol consumption (Table 4).

**Table 4:** Differences between questionnaire and biomarkers in included studies (drugs of abuse)

DRUGS OF ABUSE			
Author (reference, year)	Questionnaire (%positive result)	Biomarkers (%positive results)	Differences/Comments
Garcia Algar et al. (28) 2009	Structured questionnaire COC (1.2%); THC (1.5%) MOR (0.3) MDMA (0.1%)	COC (2.6%); THC (5.3%) MOR (4.7) MDMA (0.1%)	- Hidden non-negligible drug consumption during pregnancy.
Bessa et al. (11) (2010)	Structured questionnaire (0%)	COC (1.7%) THC (4%) COC + THC (0.3%)	- Usefulness of hair analysis for diagnosis of drug use. Significanthidden undeclared use of drugs during pregnancy
Hutson et al. (22) (2010)	Structured questionnaire COC (0.4%); THC (0.15%) AMP (1%)	COC (2%) THC (2%) AMP (8%)	- The incidence was higher than those reported through questionnaire although significance could not be determined because of near-zero self-reporting levels
García-Serra et al. (16) (2011)Friguls et al. (26) (2012)	Structured questionnaire COC (0.9%); THC (0.9%)	COC (6.4%) THC (10.3%) MDMA (0.9%) THC + COC (0.9%) THC + MDMA (0.9%) (Hair) COC (5.6%) THC (2.8%) (Meconium)	- No correlation between self-reported prevalence of illicit drug use and analytical methods - Increased sensitivity of the hair against maternal meconium in detecting exposure to cannabis. In the case of cocaine sensitivity of both matrices was similar
Joya et al. (30) (2012)	N: 347	COC (2.6%)	- Usefulness of hair analysis for diagnosis of drug use. Significant undeclared use of cocaine
Lendoiro et al. (18) (2013) (SP)	Structured questionnaire COC (4.3%); THC (2.9%) OP (1%)	COC (15.4%); THC (12.4%) OP (1%)	- The results of this study confirm the usefulness of maternal hair analysis to evidence drug use during pregnancy. - Hair analysis showed to be more sensitive than maternal interview for all drugs of abuse and medicines

## Discussion

Recent evidences support that the use of questionnaires as the only measure to investigate prenatal alcohol and drugs of abuse exposure underestimate the real prevalence. From the 15 studies comparing questionnaire versus biomarkers (8 in alcohol and 7 in other drugs of abuse) which questionnaires were compared with biomarkers, 13 of them showed an underestimated exposure by the questionnaire. This fact has been recently supported by a systematic review and meta-analysis showing that prenatal alcohol exposure as measured by meconium testing was 4.26 (95% CI: 1.34–13.57) times the pooled prevalence as measured by maternal self-reports [27]. Reasons why women don’t disclose substances of abuse consumption during pregnancy are related to shame, guilty or legal problems, especially in the US where results of drug assays have been used to terminate custody or prosecute women.

At the moment, questionnaires are widely used as they are a simple and cheap tool. However, there are no universal validated questionnaires and it has been repeatedly proved that they are not reliable and they underestimate prevalence of exposure to drugs of abuse and alcohol. Both maternal hair and meconium have been used as biological matrices in which to detect drugs of abuse consumption in pregnancy and have shown higher prevalence than clinical interviews and traditional screening methods such as blood tests and/or urine. The main advantage of these two biological matrices is that they extend the detection window considerably, as each centimetre of hair from maternal scalp corresponds to one-month period retrospectively and meconium contains the substances that the foetus has been exposed in uterus during the last two trimesters of the pregnancy. Biomarkers have been shown to be a valuable tool that could solve the problem of underreporting. On the downside, there are still a few pending questions about biomarkers to ascertain, as they can’t evaluate consumption during the first trimester nor detect very low alcohol consumptions. Some biomarkers of prenatal exposure to ethanol such as CDT and APAs can produce false positives due to its concentration increases in the third trimester of pregnancy and shows also high values in people with diabetes [28]. For instance, urine tests show limitations related to detection window and are not useful for alcohol detection. Hair tests are limited by type of hair and amounts collected, use of hair products and hair processing protocols [7]. When initial screening drug tests generate positive results, gas/liquid chromatography-mass spectrometry analysis (GC-MS or LC-MS) must be done in samples to confirm the presence of drugs in order to eliminate the false positive possibility. Some drugs as antibiotics, analgesics or antihistamines have been reported as sources of false positives so it is important to give a complete and accurate history of all prescription, OTC, and vitamin/dietary supplement/herbal drug use prior to the time of the sample collection. Despite these weaknesses, it is important to note that there are very specific and sensitive biomarkers, as PEth, the only one that detects alcohol in all patients without generating false negative and whose blood concentration correlates with the amount of ingested ethanol. The liquid chromatography coupled to tandem mass spectrometry (LC-MS-MS) is considered the most common technique to detect it in blood and urine, whereas the GC-MS is the is also frequently used in hair due to the solid nature of the matrix [29]. However, although the analytical methodology is fully developed, screening with biomarkers would be difficult to implement as it is more expensive and requires resources not available in every small clinic. The only biological screening test that is feasible for use during the pregnancy (antepartum) is urine toxicology screening. This test has many limitations. Urine screening tests can be used, but they must be confirmed by GC-MS or LC-MS tests. Recently it has been published the recommendation of using these tests in clinical settings and in our hospital we use it from a long time, not only with research purposes.

Of note, a relevant problem is the refusal of some women to accept the biomarker analysis because the results could be used to determine custody of children in case of divorce or to prosecute some of these women in countries such as USA. For these reasons, an informed consent must be required. Only in emergency cases when clinic staff determine a severe danger for the foetus, the principle of the best interest of the child will be applied.

Therefore, it can be hypothesized that biomarkers could be used as a validated confirmation when clinicians suspect of alcohol or other drugs of abuse consumption or in the context of epidemiological and clinical studies.

When clinicians find a positive for alcohol and/or drugs of abuse in biomarkers analysis not mentioned in questionnaires, different protocols promoted by public heath institutions have to be activated, focusing on the protection of the child and care, advice and help of these pregnant women to stop the use of alcohol and/or drugs.

In this review, the prevalence of consumption during pregnancy has shown to be significantly high. The highest prevalence of alcohol consumption was found the be up to 95% in the Spanish group led by Pichini and Lendorio et al. that found a prevalence of up to 15.4% positive for cocaine and 12.4% positive for THC through biomarkers [18, 30]. Prenatal exposure to alcohol and drugs of abuse increases risk not only for obstetric complications but also of lifelong consequences for the newborn. Biomarkers have shown to be the key to detect this consumption [31, 32].

The brain damage caused by the prenatal toxic exposition cannot be repaired. So, the early detection of prenatal exposure to alcohol and drugs of abuse allow these patients to benefit from early stimulation and close monitoring of their development, which will allow the implementation of timely and early interventions and therapies that are the clue to try to decrease the occurrence of secondary disabilities [5, 33].

## Conclusions

Prevalence of alcohol and drugs of abuse consumption during pregnancy is significantly high, so it is also prenatal exposure to these substances. Early detection of this exposure is essential to carry out therapeutic interventions (as in neonatal abstinence) but also to prevent deleterious effects of this exposure through early years of life.

This study has implications affecting public health programs and policies. At the moment, screening is carried out mostly through questionnaires for alcohol and drugs of abuse, but these have proved to be unreliable when compared to biomarkers. Undetected consumption could have deleterious effects over children’s health. Therefore, questionnaires underdiagnose prenatal exposure to alcohol and drugs of abuse and could promote a lack of care of the newborn. We propose the use biomarkers as the main screening tool in patients in environments with high prevalence of alcohol and drugs of abuse consumption, along with questionnaires. At the present time studies with biomarkers (maternal hair or neonatal meconium) may not be available in all services, but they should also be considered in those cases with suspected consumption although patients deny it in questionnaires.
